# ClpP-deletion impairs the virulence of *Legionella pneumophila* and the optimal translocation of effector proteins

**DOI:** 10.1186/s12866-016-0790-8

**Published:** 2016-08-02

**Authors:** Bei-bei Zhao, Xiang-hui Li, Yong-lun Zeng, Yong-jun Lu

**Affiliations:** 1 School of Life Sciences and Biomedical Center, Sun Yat-sen University, No. 135 Xingang road west, Guangzhou, 510275 China; 2 Present address: Jiangsu Information Institute of Science and Technology, Nanjing, 210042 China; 3 Present address: School of Life Sciences, Centre for Cell and Developmental Biology, The Chinese University of Hong Kong, Hong Kong, 999077 China

**Keywords:** *Legionella pneumophila*, ClpP, Virulence, T4BSS, Effectors, Substrate, Translocation

## Abstract

**Background:**

The opportunistic bacterial pathogen *Legionella pneumophila* uses substrate effectors of Dot/Icm type IVB secretion system (T4BSS) to accomplish survival and replication in amoebae cells and mammalian alveolar macrophages. During the conversion between its highly resistant, infectious dormant form and vigorously growing, uninfectious replicative form, *L. pneumophila* utilizes a complicated regulatory network in which proteolysis may play a significant role. As a highly conserved core protease, ClpP is involved in various cellular processes as well as virulence in bacteria, and has been proved to be required for the expression of transmission traits and cell division of *L. pneumophila*.

**Results:**

The *clpP*-deficient *L. pneumophila* strain failed to replicate and was digested in the first 3 h post-infection in mammalian cells J774A.1. Further investigation demonstrates that the *clpP* deficient mutant strain was unable to escape the endosome-lysosomal pathway in host cells. We also found that the *clpP* deficient mutant strain still expresses T4BSS components, induces contact-dependent cytotoxicity and translocate effector proteins RalF and LegK2, indicating that its T4BSS was overall functional. Interestingly, we further found that the translocation of several effector proteins is significantly reduced without ClpP.

**Conclusions:**

The data indicate that ClpP plays an important role in regulating the virulence and effector translocation of *Legionella pneumophila.*

**Electronic supplementary material:**

The online version of this article (doi:10.1186/s12866-016-0790-8) contains supplementary material, which is available to authorized users.

## Background

First isolated in 1977, *Legionella pneumophila*, a Gram-negative, intracellular bacterial pathogen is the agent causing the severe form of pneumonia named Legionnaires’ disease, as well as the less severe flu-like Pontiac fever [1]. It has drawn much attention for its capability of intracellular replication in both protozoa and human beings. After the endocytosis by protozoan hosts like amoebae or human alveolar macrophages, the *Legionella*-containing vacuole (LCV) inhibits phagolysosomal fusion and recruits mitochondria followed by the association of ribosome-studded membranes that later disguise LCV as endoplasmic reticulum (ER). Within this ER-like compartment, the bacterium replicates to high numbers and eventually is released through lysing the host cell for the next invasion [2].

During this process, *L. pneumophila* requires most protein products of 27 *dot/icm* (defect in organelle trafficking/intracellular multiplication) genes to constitute a type IVB secretion system (T4BSS) [2]. Although neither the composition nor the function of T4BSS has been fully understood in *L. pneumophila*, progress has been achieved in identifying and characterizing the Dot/Icm proteins. DotC, DotD, DotF, DotG and DotH comprise the core of the secretion complex which spans across the bacterial membrane. DotC and DotD are outer-membrane lipoproteins and required for DotH to target the outer membrane [3]. DotH may be the out-membrane channel through which substrates get delivered following the transit from the DotF-DotG inner-membrane proteins with the assistance of the DotL–DotM ATPase [2]. DotB, also an ATPase, interacts with DotL and may play a role in various functions such as the assembly of T4BSS, retraction of pili and/or export of substrates [4, 5]. IcmQ participates in the membrane pore formation [6], and IcmT is crucial for pore formation-mediated escape of *L. pneumophila* from protozoan or mammalian cells [7]. DotL is proposed to be a type IV coupling protein (T4CP) of T4BSS and interacts with other inner-membrane proteins including DotN, DotM and IcmS/W, a heterodimer complex functions as T4BSS adaptor, to constitute the T4CP subcomplex, a very important complex for T4BSS to facilitate substrate secretion [8, 9].

Through the T4BSS, *L. pneumophila* secretes a large number of substrate proteins called effectors that interfere with the host pathways to help bacteria evade the endosome-lysosomal pathway and replicate in host cells [2]. The effector RalF, which has guanine nucleotide exchange activity and mediates the exchange of GDP for GTP, disturbs vesicle traffic between the ER and Golgi and further promotes the biogenesis of LCV by modulating the activity and localization of the key intracellular regulator Arf1 [10]. The effector AnkB is important for the moorage of K48-linked polyubiquitinated proteins when it is anchored into the phagosome membrane by host-mediated farnesylation and interacts with the SCF1 E3 ubiquitin ligase complex. Then the K48-linked proteins are degraded and the amino acids are utilized for bacterial intracellular proliferation [11, 12]. LegK2, whose deletion causes reduced cytotoxicity, and adversely affect the intracellular survival and replication of *L. pneumophila*, acts as a protein kinase [13]. So far more than 300 effectors have been identified but many of them are considered functionally redundant, only a few are indispensable for the intracellular proliferation of *L. pneumophila*, such as MavN and SdhA [14–16].

During the shift between extracellular and intracellular environments, *L. pneumophila* encounters different growth conditions and has to respond accordingly to survive. To make the appropriate responses, *L. pneumophila* has developed a complex network to modulate the transition at different phases*.* Proteolysis has been regarded as an important and precise regulatory mechanism for both eukaryote and prokaryote to adapt to a variety of growth conditions by removing short-lived regulatory proteins, as well as misfolded and damaged proteins [17]. It is now clear that cellular proteolysis is carried out by the energy-dependent proteases such as the Lon and Clp proteases and the eukaryotic 26S proteasome [17]. To date, Clp protease is the most characterized protease in prokaryotes. It consists of two functional subunits: a cylinder-like proteolytic core named ClpP which is widely distributed and highly conserved, and two chaperone rings with ATPase activity such as ClpA, ClpC, ClpE or ClpX [17, 18]. The protease core consists of 14 ClpP serine peptidase subunits stacked in two heptameric rings, forming an internal chamber in which the active sites are sequestered from the cytoplasm [19]. The Clp ATPases are responsible for the recognition, unfolding and translocation of substrates into the degradation chamber [20].

It is widely accepted that Clp proteases are involved in many physiological processes of bacteria. In a range of low GC Gram-positive bacteria including *Bacillus subtilis, Listeria monocytogenes* and *Lactococcus lactis*, ClpP-deficient mutants suffer restricted growth at high temperatures [21–24]. ClpP is also considered as the major determinant for the turnover of bulk proteins in *B. subtilis* at the transition from exponential stage to competence and further sporulation stages [24, 25]. Moreover, both ClpP and its ATPase chaperones play significant roles in virulence expression and regulation in various bacterial pathogens. For instance, *S. aureus* cells lacking the ClpB chaperone are unable to replicate intracellularly in bovine cells [26]. The absence of ClpP in *L. monocytogenes* results in the lack of listeriolysin O, a major virulence factor implicated in phagosome lysis [23, 27]. Our recent research has shown that ClpP is required for the transmission traits of *Legionella pneumophila* during the transition in its biphasic life cycle, including some traits associated with virulence such as cytotoxicity and intracellular proliferation in the amoebae host *Acanthamoeba castellanii* [28]. In this report, studies were focused on the function of *Legionella pneumophila* ClpP in the mammalian cell J774A.1 and results revealed that the deletion of *clpP* severely impaired the bacterial virulence and the translocation of several T4BSS effectors though the functional integrity of T4BSS was not fully neutralize.

## Results

### ClpP is essential for intracellular proliferation of *L. pneumophila* in macrophages

**Fig. 1:**
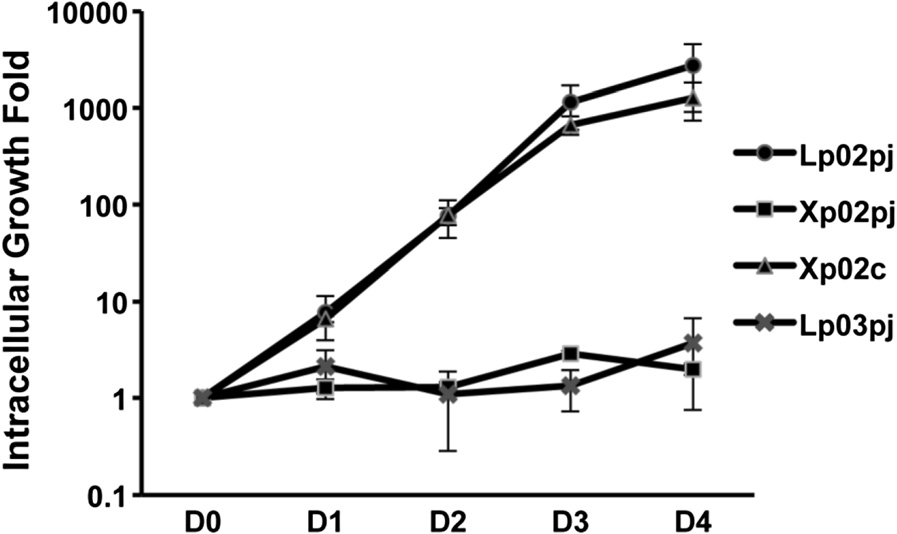
Intracellular growth of *clpP*-deficient *L. pneumophila* strain Xp02 in J774A.1 was impaired. J774A.1 cells were seeded in 24-well plates and infected with *L. pneumophila* at an MOI of 1. At each time point, cells were lysed and the CFU was determined by plating dilutions onto BCYE plates. The intracellular growth kinetics of Lp02 (●), *clpP* mutant (■), *clpP* complemented strain (▲), and *dotA* mutant Lp03 (X) were shown. Points indicate mean values and error bars indicate standard deviations of three independent experiments

We have shown previously that ClpP is essential for intracellular multiplication of *L. pneumophila* in amoebae *A. castellanii* [28]. To investigate whether ClpP also affects intracellular proliferation of *L. pneumophila* in macrophages, the wild type, the *clpP*-deficient mutant, the constitutive complementation, and the Dot/Icm-deficient *dotA* mutant strains were grown to the stationary phase and used to infect *Mus musculus* macrophages. The infection was allowed to proceed for 5 days and the intracellular proliferation was evaluated by plating the cell lysate onto BCYE plates per 24 h. As shown in Fig. 1, the wild type Lp02pj and the complemented strain Xp02c exhibited essentially identical proliferation rate in J774A.1 cells. In contrast, both the *clpP*-deficient mutant Xp02pj and the *dotA* mutant strain Lp03pj showed significantly impaired multiplication. These results indicate that ClpP is essential for the intracellular growth of *L. pneumophila* in macrophage.

### *clpP* mutant is degraded rapidly upon entry into the host cells

**Fig. 2:**
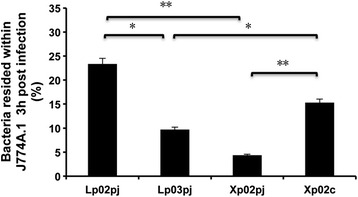
The *L. pneumophila clpP* mutant Xp02 was degraded within 3 h of phagocytosis. J774A.1 cells were seeded in 24-well plates and infected with *L. pneumophila* at an MOI of 1. After 3 h, the survival of bacteria was determined by plating dilutions onto BCYE plates and calculating the numbers of CFU of *L. pneumophila* lysed from infected J774A.1. *, *p* < 0.05; **, *p* < 0.01. The phagocytosis assay was carried out in triplicate. Shown are the averages and standard deviations of three independent counts

Avirulent *L. pneumophila* strains decrease rapidly in the first hours of phagocytosis [29]. Because the *clpP-*deficient strain showed severely reduced cytotoxicity and intracellular replication (Fig. 1 and [28]), it is of interest to explore whether ClpP is essential for preventing *L. pneumophila* from degradation after uptake into host cells. To this end, J774A.1 were exposed to the stationary-phase *L. pneumophila* strains and co-cultures were maintained for 3 h before the cells were lysed. The significantly lower percentage (*p* < 0.01) of bacteria residing within host cells 3 h post infection (Fig. 2) demonstrated the impaired survival capability of *clpP*-deficient mutant after phagocytosis. As a negative control, the *dotA*-deficient mutant Lp03 similarly exhibited impaired survival capability compared with the wild type strain Lp02 (Fig. 2). These data indicate the rapid degradation of the *clpP*-deficient bacteria post infection and suggest that *clpP* mutant cannot escape the host defense systems.

### The *clpP* mutant fails to evade the late endosome-lysosomal pathway

**Fig. 3:**
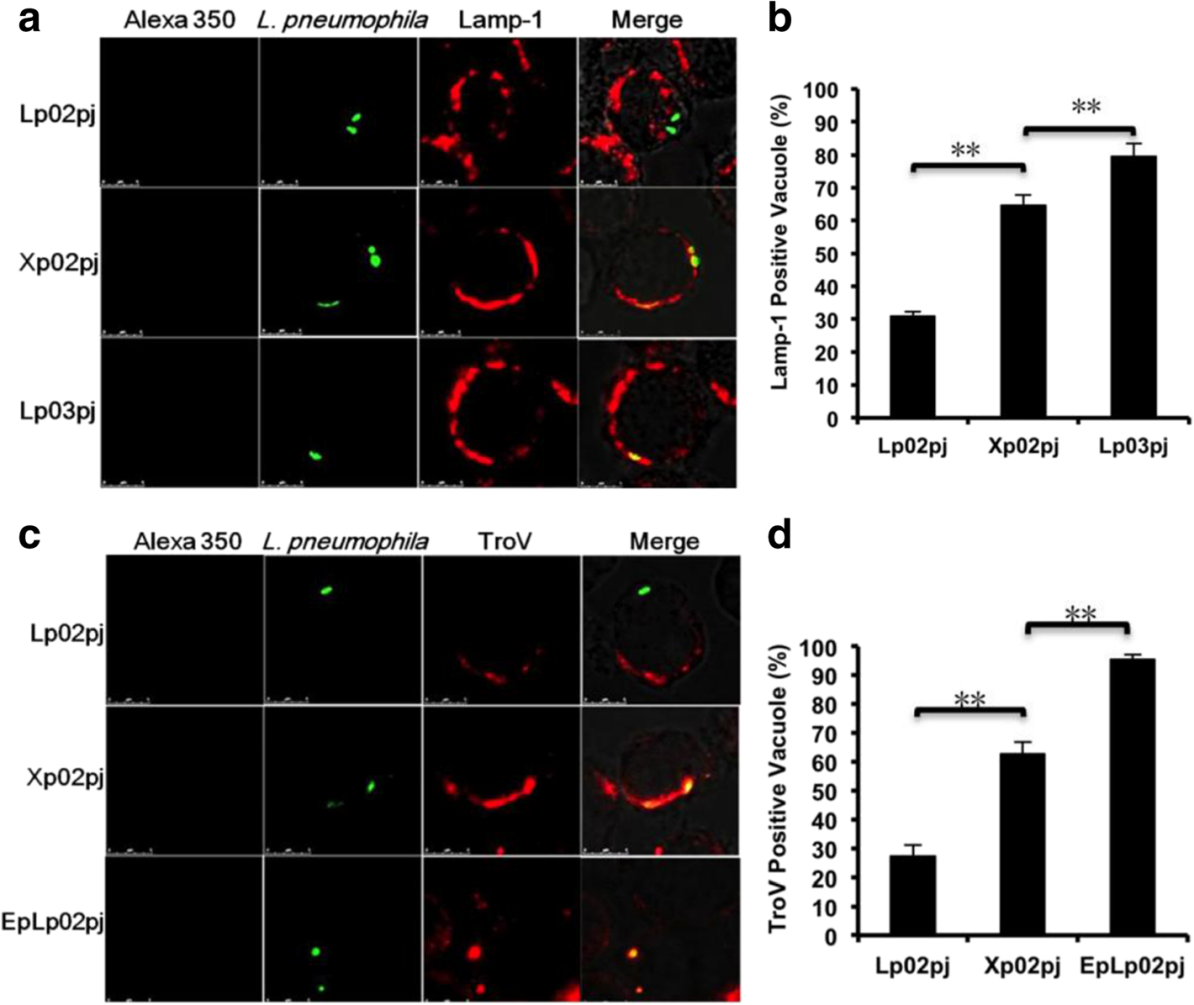
Immunofluorescence analysis of the late endosome and lysosome in macrophages infected with *L. pneumophila.* J774A.1 cells were incubated with the wild-type *L. pneumophila* or mutant strains for 2 h, then fixed and stained with a monoclonal antibody specific to LAMP-1 or TroV to identify the macrophage late endosomes or lysosomes. **a** Phagosomes containing wild-type strain Lp02pj were not co-localized with late endosomes, whereas phagosomes containing Xp02pj or Lp03pj were stained and colocalized with LAMP-1. **b** Meanwhile the percentages of *L. pneumophila*-containing phagosomes fused with the late endosomes were determined. **, *p* < 0.01. **c** The fusion of phagosomes with lysosomes was also examined using confocal microscopy and **d** quantified as above. **, *p* < 0.01. The immunofluorescence assay was carried out in triplicate. Shown are the averages and standard deviations of three independent counts. The number of J774A.1 cells for each count is about 100

To assess whether the *clpP*-deficient mutant could survive the endosome- lysosomal pathway, the molecular morphology of phagosomes containing *L. pneumophila* was investigated in J774A.1. Lysosomal-associated membrane protein 1 (LAMP-1) and the Texas-red conjugated ovalbumin (TroV) were utilized as markers of the late endosome and lysosome, respectively. The wild type strain Lp02pj that was grown in broth until it reached exponential phase was used as the negative control (labeled as EpLp02pj in Fig. 3c and d) for the lysosome fusion assay because the phagosomes containing Dot/Icm-deficient *dotA* mutant strain do not acquire lysosomal TroV in the first several hours post-infection [30]. Predictably, the wild type strain Lp02pj that can replicate in host cells was excluded from LAMP-1 stained compartments, while the *clpP*-deficient mutant Xp02pj was frequently colocalized with LAMP-1 staining, just like the negative control Lp03pj (Fig. 3a). Staining results showed that 31 % of the Lp02pj-containing phagosomes exhibited detectable LAMP-1 accumulation, whereas 65 % of the Xp02pj-containing phagosomes were LAMP-1 positive. Although higher than that of Lp02pj-containing phagosomes (*p* < 0.01), the percentage of LAMP-1 positive Xp02pj-containing phagosomes was still significantly lower than that of Lp03pj-containing phagosomes (*p* < 0.01) (Fig. 3b). Similar results were obtained in the lysosome fusion assay in which the percentages of TroV positive phagosomes containing Lp02pj, Xp02pj or EpLp02pj were about 27, 63 or 95 %, respectively (Fig. 3c and d). These means were significantly different from each other (*p* < 0.01). Based on these findings, we conclude that ClpP is important for *L. pneumophila* to escape the late endosome-lysosomal pathway of mammalian host cells.

### Loss of ClpP does not affect the expression of *dot/icm* components

**Fig. 4:**
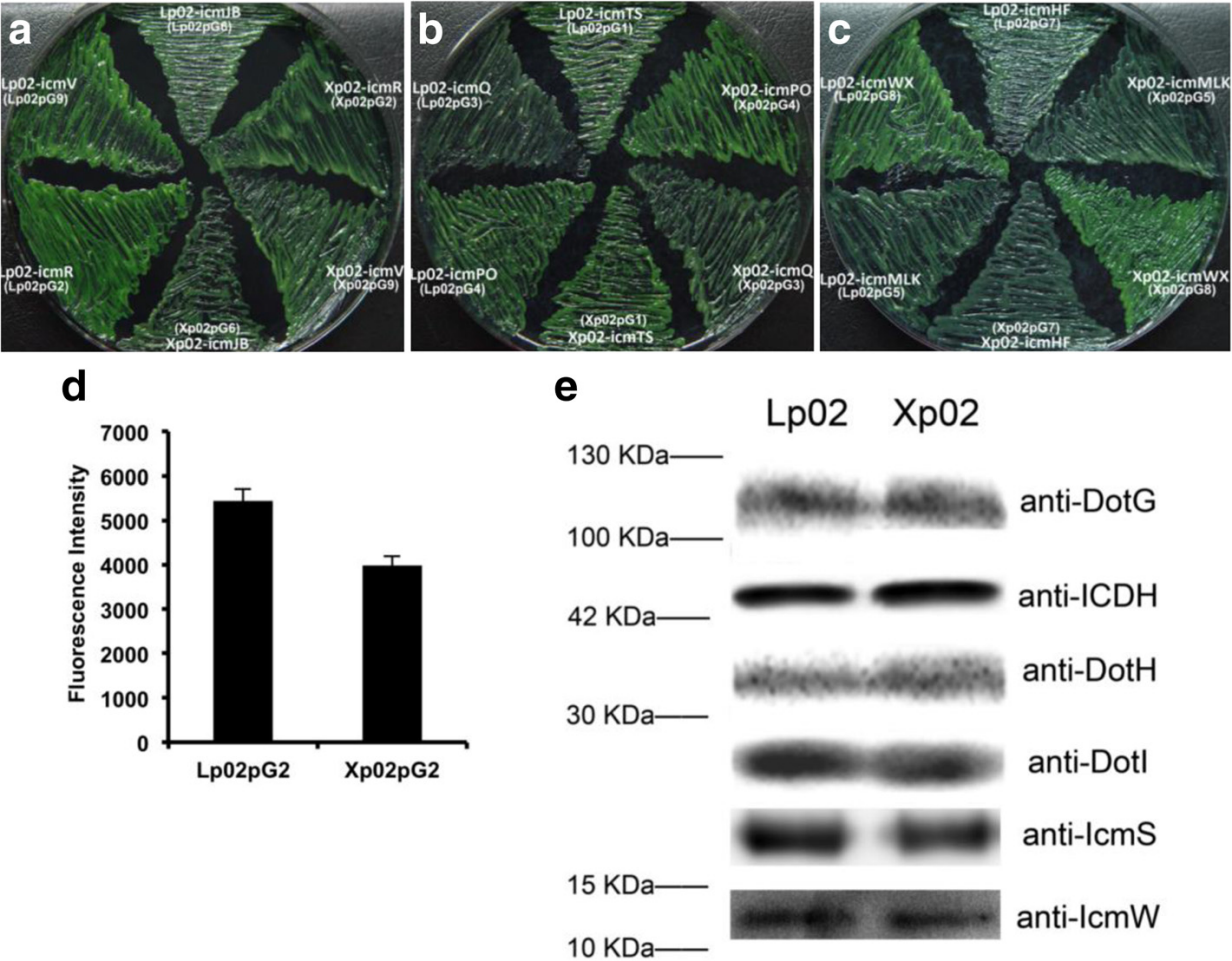
Analysis of the promoter activitiy and the expression of the *dot/icm* components. **a**-**c** Promoter activities of 9 *dot/icm* operons were examined through streaking and comparing the bacterial strains harboring the promoter-*gfp* fusion plasmids on plates. **d** Quantified fluorescence intensities of the wild-type Lp02 and the *clpP* mutant Xp02 harboring *icmR:gfp* fusion plasmid. There is no statistical difference between the two samples. **e** Western blot analysis of the expression of DotH, DotI, DotG, IcmS and IcmW in the wild type and the *clpP* deficient mutant strain. A western blot using antibody to isocitrate dehydrogenase (ICDH) was used to confirm even protein levels of the samples. Shown are the averages and standard deviations of 2 to 3 independent experiments, each performed in triplicate

To survive and multiply within phagocytic host cells, *L. pneumophila* manipulates host cellular processes, alters the host endocytic pathway, thereby inhibits rapid phagosome-lysosome fusion and creates a niche for its replication. These are achieved with the help of Dot/Icm T4BSS and effectors [2, 14]. In view of this, we presumed it might be the abnormal function of T4BSS or interrupted secretion of effectors that caused the impaired survival and intracellular proliferation of the *clpP*-deficient strain in host cells. To test this hypothesis, we measured the transcription activity of *dot/icm* genes in 9 operon areas [31] by examining the promoter activities of these genes. Promoters were fused with *gfp* reporter gene and transformed into both Xp02 and Lp02. The fluorescence intensities, as well as the expression of DotH (IcmK), DotI (IcmL) and DotG (IcmE), which have been proved to be components of the Dot/Icm system [3], were measured. Plate patching results showed that there were no significant differences in fluorescence intensities between Lp02 and Xp02 harboring the *icm-gfp* fusion plasmids (Fig. 4a-c). The fluorescence in the liquid culture confirmed the results of patching and only a slight reduction of fluorescence intensity (*p* > 0.05) was found in the *icmR-gfp*-harboring Xp02pG2, compared to that in Lp02pG2 (Fig. 4d). Furthermore, Western blot analysis indicated that there were no differences between the expression levels of DotH, DotI, DotG, IcmS and IcmW in Lp02 and Xp02 (Fig. 4e). Taken together, these results indicate that loss of ClpP might not significantly influence the expression of the T4BSS components of *L. pneumophila.*

### The T4BSS secretion apparatus is still functional in the *L. pneumophila clpP* deficient mutant

**Fig. 5:**
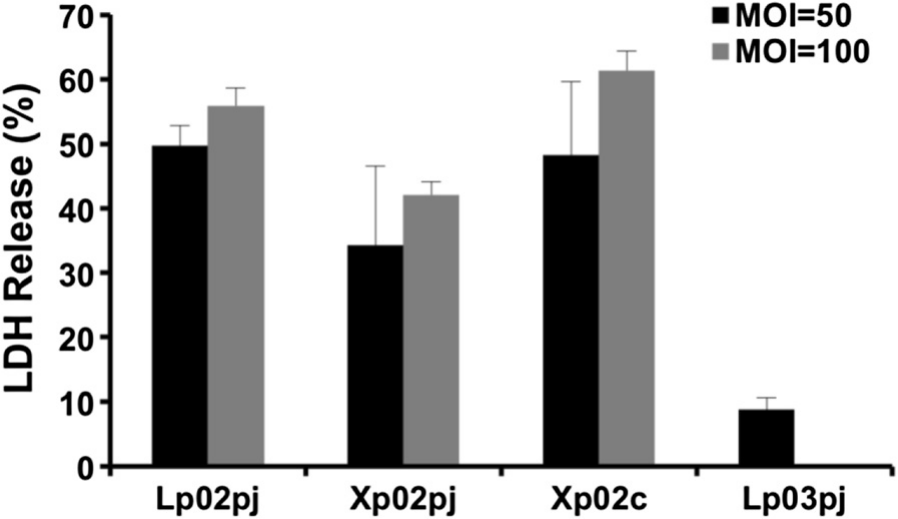
The *clpP*-deficient *L. pneumophila* still has a functional T4BSS. Contact-dependent cytotoxicity of the wild-type strain Lp02, the *clpP* mutant Xp02, the complemented strain Xp02c and the *dotA* mutant Lp03. Infection was performed at an MOI of 50 and 100, and cell permeabilization was detected by the release of the intracellular enzyme lactate dehydrogenase (LDH). Shown are the averages and standard deviations of three independent experiments, each performed in triplicate (duplicate for cytotoxicity assays). And there is no statistical difference between the strains

The Dot/Icm T4BSS system of *L. pneumophila* consists of 27 proteins [2]. Because not all antibodies to these components are available, we cannot determine whether the T4BSS of the *clpP* deficient mutant still functions normally through Western blot analysis. To investigate the integrity of T4BSS complex in the *clpP* mutant, we utilized contact-dependent cytotoxicity assay. It is well comprehended that *L. pneumophila* triggers contact-dependent cytotoxicity, which is actually caspase-1-mediated or caspase-3-mediated cell death, through delivering flagellin protein into host cells and this process requires a functional Dot/Icm T4BSS complex [32, 33]. *L. pneumophila* strains harboring pJB908 were grown to post-exponential phase in broth and co-cultured with J774A.1 at an MOI of 50 and 100. After incubation for 2 h, the permeabilization of cells was detected by the release of the intracellular enzyme lactate dehydrogenase (LDH). At an MOI of 50, both the wild type and the complemented strains showed high contact-dependent cytotoxicity and the LDH release was 50 and 48 %, respectively. In contrast, the LDH release of the *dotA*-deficient strain Lp03 was only 9 %. Intriguingly, the LDH release of the *clpP* mutant was 34 % and remained cytotoxic, although it was lower than that of the wild type and the complemented strains (*p* > 0.05) (Fig. 5). Similar results were observed when the MOI was raised to 100 (Fig. 5). These findings imply that the T4BSS complex in the *clpP* mutant may be still functional and overall integrated.

### ClpP controls the translocation efficiency of some T4BSS effectors

**Fig. 6:**
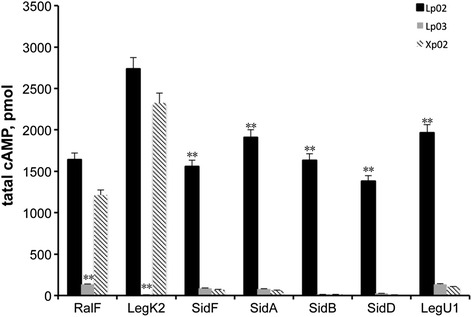
*clpP*-deficiency impaired the translocation of some effectors. J774A.1 cells were infected with the wild type (Lp02), the *dotA* mutant (Lp03) and the *clpP* mutant (Xp02) *L. pneumophila* strains expressing the indicated Cya hybrid proteins. After 1 h of infection, tissue culture cells were lysed and cAMP was extracted from the sample. Total cAMP production induced by translocation of the hybrid was quantified using an enzyme-immunoassay system, indicated as pmol. Results represent average values of experiments performed in triplicate. **, *p* < 0.01

Since the T4BSS apparatus is still functional in the *clpP* mutant, we therefore examined if the translocation frequency of effectors is impaired. A well-established reporter gene for translocation assay, *cya*A [34], was fused with seven effector coding genes, respectively. The resulting plasmids were transformed into the wild type Lp02, the *clpP* deficient strain Xp02 and the *dotA* mutant strain Lp03, respectively. The recombined strains were then infected J774A.1 cells and the fused genes were expressed under IPTG-induction for translocation frequency assay. As shown in Fig. 6, the cAMP levels of RalF and LegK2 showed no difference between Lp02 and Xp02, respectively, whereas the cAMP levels of SidA, SidB, SidD, SidF and LegU1 in Xp02 were significantly lower than that in Lp02, which decreased to 3.27, 0.74, 7.64, 0.25 and 6.06 % of that in Lp02, respectively. Immunoblotting assay showed that all Cya fusion proteins were expressed abundantly (Additional file 1: Figure S1). These results indicate that the translocation of some T4BSS effectors is partly controlled by ClpP.

## Discussion

In the current study, we found that the *L. pneumophila clpP* deficient mutant exhibited poor survival and intracellular multiplication in J774A.1 cells (Figs. 1 and 2). Furthermore, the mutant strain could not escape the late endosome-lysosomal pathway (Fig. 3). Thus, consistent with our previous results obtained in the amoebae host *A. castellanii* [28], ClpP may be required for the expression of virulence in *L. pneumophila.* To investigate how ClpP regulates the virulence, we tested whether the deficiency of *clpP* could affect the component expression and the function of T4BSS complex. The 27 proteins of *dot/icm* components may not be significantly affected based on the results of transcriptional activity assay together with immunoblotting analysis (Fig. 4). Although we did not examine the expression of all 27 proteins, the findings that the *clpP* mutant could still induce contact-dependent cytotoxicity against host cells (Fig. 5), together with that both RalF and LegK2 could be translocated into host cells, indicated that the T4BSS function was not compromised seriously in the absence of ClpP. However, the fact that the secretion of some effectors was impaired in the *clpP*-deficient strain suggested that one of the strategies for ClpP to affect the virulence of *Legionella pneumophila* is *via* regulating the translocation of effectors.

The phenotype of *clpP*-deficient *L. pneumophila* resembles that of the IcmS/W mutants, the T4BSS chaperone. They all exhibit significantly impaired intracellular replication, but still maintain fair contact-dependent cytotoxicity against host cells (Figs. 1 and 5) [35, 36]. Moreover, the translocation of three IcmS/W-mediated effectors (SidA, SidB and SidD) and LegU1 was impaired obviously in the absence of ClpP, and the translocation of non-IcmS/W-mediated RalF was unchanged without ClpP (Fig. 6) [37]. More interestingly, the non-IcmS/W-mediated effector SidF showed reduced translocation efficiency in *clpP*-deletion mutant (Fig. 6), which was similar in *icmS/icmW* double mutant, and the translocation of SidA, SidB and SidD in *clpP*-deletion mutant was more neutralized than that in *icmS* or *icmW* single mutant [37]. Taken together, a hypothesis that *clpP*-deletion mutant might resemble an absolutely IcmSW-abolishing mutant could be proposed. Currently, how ClpP affects IcmS/W subcomplex is difficult to be clarified because the expression level of each protein in *clpP*-deletion mutant is unchanged (Fig. 4e). Previous studies have shown that ClpP affects the virulence expression in some gram-positive pathogens such as *Staphylococcus aureus*, *Streptococcus pneumoniae* and *L. monocytogenes* [23, 26, 38, 39]. More details about the virulence regulation by ClpP have been revealed in *Salmonella enterica* serovar Typhimurium and *Yersinia pestis*, where ClpP governs the protein levels of important virulence-regulating factors for type III secretory systems (T3SS), RpoS and YomA [40, 41]. For pathogens containing T4BSS, little information about the association between Clp proteases and other virulence-related factors is available. Recently studies on the DotL-IcmSW coupling subcomplex of *L. pneumophila* T4BSS have revealed that ClpAP protease is responsible for the degradation of DotL in the absence of IcmS. Briefly, in the absence of IcmS/W mediation, abundant effector proteins could be trapped within DotL, and subsequently the jamming complex is subjected to specific degradation by ClpAP [8, 42]. Interestingly, although DotL degradation by ClpP requires specific recognition by ClpA, the single deletion of *clpA* does not affect the intracellular replication or the protein level of DotL [8]. Thus, it is possible that ClpP may affect DotL-IcmSW-mediated effector translocation through other processes bypassing ClpA. On the one hand, ClpP may influence the optimal assembly of T4BSS rather than protein expression, especially in DotL-IcmSW coupling subcomplex. On the other hand, successful effector translocation mediated by IcmS/W might need essential cleavage or modification by ClpP protease. In the second situation, translocation signal peptides may be involved. Up to now, a C-terminal signal peptide has been proven to be essential for the translocation of nearly all T4BSS effectors and an internal signal sequence has been found to be important in IcmS/W-mediated translocation [43, 44]. Thus, the latter signal peptide may be the possible target of ClpP modification or regulation. In our future proposal, the association of ClpP with the internal signal peptide in IcmS/W-mediated effectors needs to be explored, and we also hope to examine the assembly status of the DotL-IcmSW subcomplex in *clpP*-deletion mutant.

It is also possible that the neutralized intracellular replication is partially due to the impaired stress tolerance of *clpP*-deficient *L. pneumophila.* In both natural aquatic and intracellular environment, *L. pneumophilla* would encounter various stresses [45, 46]. Legitimately, a rapid responding system involving proteolytic procedures would be beneficial to the stress tolerance of *L. pneumophilla*. Studies have revealed that ClpP protease plays significant roles in DNA repair and other stress responses and helps bacteria adapt to many harsh conditions [38, 47–49]. Likewise, *L. pneumophila* ClpP is required for the stationary-phase resistance against various stresses such as oxidation, acidity, osmotic stress and inappropriate temperatures [28]. Among these stresses, acid resistance might be the most critical in *Legionella*’s intravacuolar survival. *L. pneumophila* could neutralize the acidic environment of phagosome and subsequently maintain a nearly neutral-pH vacuole during the first hours of uptake [50]. However, about 18 h later *L. pneumophila* would still encounter the acidic environment when the LCV mature into low-pH and endocytic compartments [51]. Considering our previous finding that the *clpP* mutant strain exhibited reduced acid resistance and vulnerable cell surface compared to the wild type strain [28], we wonder whether and how much the “weakness” the *clpP* mutant displays under unfavorable environments contributes to the disability of virulence expression, this assumption needs deeper exploration.

## Conclusion

In this study, our data show that the loss of *clpP* prevents *L. pneumophila* from evading the endocytic pathway and replicating in host cells. Our results also revealed that the *clpP*-deficiency affects the translocation of some T4BSS effectors without impairing the integrity of T4BSS. Taken together, *L. pneumophila* ClpP is an indispensable factor for the virulence and the translocation of some T4BSS effectors.

## Methods

### Bacterial strains, cells and reagents

The bacterial strains, plasmids and primers used in this work are listed in Tables 1, 2 and 3, respectively. *L. pneumophila* strains were cultured on buffered charcoal yeast extract (BCYE) plates, or in N-(2-acetamido)-2-aminoethanesul-fonic acid (ACES)-buffered yeast extract (AYE) medium [52], supplemented with 5 μg/ml chloramphenicol when necessary. *Escherichia coli* strains were cultured on Luria-Bertani (LB) agar plates, or in LB broth, supplemented with 30 μg/ml chloramphenicol or 100 μg/ ml ampicillin when necessary. *A. castellanii* (ATCC 30234) was grown in proteose yeast extract glucose medium (PYG) at 30 °C [53]. J774A.1 (ATCC TIB-67) cells were maintained in Roswell Park Memorial Institute 1640 (RPMI1640) medium supplemented with 10 % fetal bovine serum (FBS) (Gibco), at 37 °C and in 5 % (v/v) CO_2_. Bacto yeast extract and proteose peptone were obtained from Becton Dickinson Biosciences. All other reagents were purchased from Sigma Co., unless specified otherwise.

**Table 1:** Bacterial strains used in this study

Strain	Characteristics	Reference or source
*E.coli* DH5α	host strain used for cloning	Lab collection
Lp02	Virulent *L. pneumophila* serogroup 1, strain Philadelphia, *rpsL, HsdR* ^*−*^ *, Thy* ^*−*^	Lab collection
Lp03	Virulent *L. pneumophila* serogroup 1, strain Philadelphia*, dot03*, *rpsL, HsdR* ^*−*^ *, Thy* ^*−*^	[58]
Xp02	Lp02 with *clpP* deletion	This study
Xp02c	Xp02 containing pXL901 for complementation	This study
Lp02pj	Lp02 containing pJB908	This study
Lp03pj	Lp03 containing pJB908	This study
Xp02pj	Xp02 containing pJB908	This study
Lp02pGX*	Lp02 containing plasmids pGB908X* with *icm* promoter-gfp fusions	This study
Xp02pGX*	Xp02 containing plasmids pGB908X* with *icm* promoter-gfp fusions	This study
Lp03pG9	Lp03 containing plasmid pGB9089 with *icmV* promoter-gfp fusion	This study

The X* represents the numbers of the plasmids or bacterial strains containing the *icm* promoter-*gfp* fusions: 1. *icmTS*, 2. *icmR*, 3. *icmQ*, 4. *icmPO*, 5. *icmMLKEGCD*, 6. *icmJB*, 7. *icmHF*, 8. *icmWX*, 9. *icmV*

**Table 2:** Plasmids used in this study

Plasmids	Characteristics	Reference
pBRDX	Suicide delivery vector, *rdxA sacB* Cm	[54]
pBR*ΔclpP*	pBRDX::*clpP* for *clpP* deletion	This study
pJB908	Insert t*hy* gene, mutate *mob* into pkB5	[4]
pBC(gfp)Pmip	ColE1 *ori* Cm P*mip gfpmut2*	[59]
pZL01	Insert *SacI/PstI* fragment encoding GFP, P*mip* into pJB908	This study
pXL901	Complementation plasmid, derived from pZL01, with *gfpmut2* changed for *clpP*	This study
pTLpflaG	Insert *BamHI/SphI* fragment encoding GFP, *flaA* promoter into pTLP6	[57]
pGB908	Insert *XbaI/SphI* fragment of *gfpmut3* into pJB908	This study
pGB908X*	Insert 9 *icm* promoters of operon areas into pGB908	This study
pCyaSidJ	Insert *EcoRI/SalI* fragment of CyaA catalytic domain and SidJ into pKB5	[34, 50]

The X* represents the numbers of the plasmids or bacterial strains containing the *icm* promoter-*gfp* fusions: 1. *icmTS*, 2. *icmR*, 3. *icmQ*, 4. *icmPO*, 5. *icmMLKEGCD*, 6. *icmJB*, 7. *icmHF*, 8. *icmWX*, 9. *icmV*

**Table 3:** Primers used in this study

Primer	5’ → 3’ sequence	Source
XP-F1	TGGTCCGGATCCCTGCCAGTAGGTCCTATAAG	This study
XP-P1	TATGACATACAAGTTGCTGGACATTCTAC	This study
XP-F2	CAACTTGTATGTCATAGGAACGCTCACC	This study
XP-P2	TGGTCCAGATCTTGGGAAAATTGACAAACCGT	This study
XL091F	TGGTGGTCTAGATTTAAGAAGGAGATATACATATGCCAGGCTATTCAGATAAT	This study
XL091R	TGGTGGGCATGCTTATAAGTCTGTAGAATGTCCAGC	This study
Rac-F	CGGGATCCATGCATCCAGAAATTGAAAAGGC	This study
Rac-R	ACGCGTCGACTTATTTCTTATAACTCGATCTACTTTC	This study
LK2c-F	CGGGATCCATGGTTTATTACATAAATTTGAAGGAACAA	This study
LK2c-R	ACGCGTCGACTTAGCTTGGGCCTCGCATCA	This study
SDAc-F	CGGGATCCTTGGTATATTATGAGATCATTAAGGATAT	This study
SDAc-R	ACGCGTCGACTTAAATAGTAAGACTCGAGTTAGTTG	This study
SDBc-F	CGGGATCCATGGCTAAAATTTATAATGCACCAAAAC	This study
SDBc-R	ACGCGTCGACCTAATTTATTTCTGGTATACTTTTTACG	This study
SDDc-F	CGGGATCCTTGGTATATTATGAGATCATTAAGGATAT	This study
SDDc-R	ACGCGTCGACTTAAATAGTAAGACTCGAGTTAGTTG	This study
SDFc-F	CGGGATCCATGCCACGAATCACTGAAAATAT	This study
SDFc-R	ACGCGTCGACTTAGAAGTTTACTGGCGTGG	This study
LU1c-F	CGGGATCCATGAAAGCAAAATACGAC	This study
LU1c-R	ACGCGTCGACCTACAATGGCTCACATTGGC	This study

### DNA manipulation, chromosomal in-frame deletion, complementation assay

All DNA manipulations were performed according to standard protocols and the in-frame deletion was carried out as described before [28] except that the suicide vector was substituted for pBRDX and the *L. pneumophila* strain for Lp02. Briefly, the upstream and downstream flanking sequences of *clpP* were amplified by PCR using P_XP-F1_/P_XP-R1_ and P_XP-F2_/P_XP-R2_ primer pairs, respectively. The PCR products were mixed as templates for the subsequent fusion PCR using P_XC-F1_/P_XC-R2_ as the primer pair. The Fusion PCR product was digested with *Bam*HI and *Bgl*II, and sub-cloned into the pBRDX vector [54], yielding pBR*ΔclpP*. Then, pBR*ΔclpP* was introduced into the wild-type Lp02 strain by electroporation and chloramphenicol-resistant colonies were selected on BCYE-Cm plates. Transformants were patched and inoculated into AYE broth and then spread on BCYE plates containing 20 μg/ ml metronidazole. Bacteria were cultured for about 3 days at 37 °C to screen for strains without the suicide vector. Positive colonies were verified by PCR and sequencing.

In the complementation assay, a RSF1010 pKB5-derived vector pJB908 was utilized as the cloning backbone [4]. The ColE1-type plasmid pBC(gfp)Pmip, which had been used as a complementation vector previously [28], was digested with *Sac*I and *Pst*I. The fragment was ligated with pJB908, yielding pZL01. Then, the sequence of *clpP* was amplified using P_XL091F_/P_XL091R_ and digested with *Xba*I and *Sph*I. Finally, the digestion product was ligated with pZL01 to construct pXL091, in which Pmip controlled the transcription of *clpP* gene constitutively. Because the resulting plasmid pXL091 contains the thymidylate synthetase gene originating from pJB908 backbone, transformants harboring pXL091 could be easily selected on BCYE plates devoid of thymidine, without addition of any antibiotics.

### Phagocytosis assay and intracellular growth assay

For phagocytosis, J774A.1 cells were seeded into 24-well tissue culture plates (2.5 × 10^5^ cells per well) and allowed to adhere overnight. *L. pneumophila* strains harboring pJB908 were grown to stationary phase, which were predominantly motile (OD_600_ 3.7–4.5), and used for infection at a multiplicity of infection (MOI) of 1 after suspension in culture medium. The infection was synchronized by centrifugation at 500 g for 10 min and incubating for 30 min. The extracellular bacteria were removed by washing 3 times. After another 3 h of incubation, cells were lysed with 0.05 % saponin for plating dilutions onto BCYE plates and colony forming unit (CFU) counting. The percentages of bacteria resided within host cells were calculated as below: Phagocytosis percentage (%) = 100 x (CFU of cell lysate after incubating for 3 h)/(CFU of bacterial suspensions added at the initiation of infection).

The intracellular growth assay in J774A.1 cells was performed using a similar protocol as the assay in *A. castellanii* [28]*,* except that the cell culture medium and the washing buffer were replaced with pre-warmed RPMI1640, the MOI was reduced from 10 to 1, and the lysing reagent was changed to 0.05 % saponin.

### Contact-dependent cytotoxicity assay

J774A.1 cells were seeded into 96-well plates (1.0 × 10^5^ cells/well) 24 h prior to infection. *L. pneumophila* strains harboring pJB908 were grown to post-exponential phase (OD_600_ 3.0–3.7) in AYE broth, then harvested and used to infect J774A.1 cells at an MOI of 50 and 100. The culture plates were centrifuged at 400 g for 10 min and incubated for 2 h at 37 °C. After incubation, the cytosolic enzyme lactate dehydrogenase (LDH) release of supernatants was determined using the CytoTox-ONE 96 cytotoxicity assay kit (Promega), according to the instructions provided by the manufacturer. The level of *Legionella*-induced contact-dependent cytotoxicity was calculated as below: LDH release (%) = 100 x (experimental – culture medium background)/(maximum LDH release – culture medium background). Maximum LDH release was the LDH release from cells treated with 0.9 % Triton X-100, used as a positive control.

### Cya translocation assays

To construct the CyaA fusions, the sequences of *ralF, legK2, sidA*, *sidB, sidD, sidF and legU1* were amplified using the primers P_RAc-F_/P_RAc-R_, P_LK2c-F_/P_LK2c-R_, P_SDAc-F_/P_SDAc-R_, P_SDBc-F_/P_SDBc-R_, P_SDDc-F_/P_SDDc-R_, P_SDFc-F_/P_SDFc-R_ and P_LU1c-F_/P_LU1c-R_ respectively. Then the DNA products were digested with *Bam*HI and *Sal*I and ligated into the *Bam*HI/*Sal*I site of a pJB2581-derived plasmid with *cyaA*-*sidJ* fusion [34, 50], yielding pCA1-pCA7.

Cya translocation assay was carried out as described before [55]. Briefly, J774A.1 cells were seeded into 96-well plates (2.5 × 10^5^ cells/well) and infected with *L. pneumophila* strains expressing CyaA fusion proteins at an MOI of 30. After incubation for 1 h at 37 °C, cells were washed and lysed, and total cAMP was extracted and determined using cAMP Enzyme Immunoassay Kit (Sigma-Aldrich). Creation of the fusion proteins was assessed by Western blotting using a monoclonal antibody to CyaA (CyaA (3D1), Santa Cruz Biotechnology).

### Immunoblotting

*L. pneumophila* bacteria were harvested by centrifugation and washed in pre-cooled sterile water for 3 times. Then the samples were resuspended in 1 × Laemmli Sample Buffer, boiled for 10 min and centrifuged at 13,000 rpm for 10 min at 4 °C. Supernatants were then collected and loaded on SDS-polyacrylamide gels for analysis. The primary antibodies used and their dilutions were as follows: rabbit Anti-DotH, DotG and DotI (1:750; gifts from Dr. Luo ZQ), rabbit Anti-IcmS, IcmW and ICDH (1:200, 1:1000 and 1:2000; gifts from Dr. Vogel JP). The secondary antibodies were horseradish peroxidase (HRP)-conjugated goat anti-mouse or goat anti-rabbit (1:10000; Sigma). SuperSignal West Pico (Pierce) was used for signal detection.

### Interaction of phagosomes with the endocytic pathway

The fusion of *Legionella*-containing phagosomes with the late endosome was assessed by detecting the co-localization of phagosomes with the lysosome-associated membrane protein 1 (LAMP-1) [52]. J774A.1 cells were seeded into 24-well tissue culture plates with 12-mm coverslips (1 × 10^5^ cells/well) and incubated overnight at 37 °C. Then the cells were infected with *L. pneumophila* harboring pJB908 (OD_600_ 3.7–4.5) at an MOI = 5. After incubation for 1 h, extracellular bacteria were eliminated by treating with 100 μg/ml gentamicin sulfate for 0.5 h. Then the coverslips were washed, transferred to clean wells, and fixed with 4 % paraformaldehyde solution for 10 min. To label extracellular bacteria, cells blocked with PBSSG (PBS containing 5 % sucrose and 2 % goat serum) buffer for 1 h were loaded with mouse anti-*Legionella* primary antibody (1:150; Santa Cruz Biotechnology), followed by Alex Fluo 350 conjugated goat anti-mouse IgG secondary antibody (1:2000; Molecular Probes). After being permeabilized with cold methanol for 20 s, intracellular bacteria were also blocked and labeled as above, with secondary antibody changed to the Alex Fluo 488 conjugated goat anti-mouse IgG. Finally, cells were loaded with rat anti-mouse Lamp-1 primary antibody (1:150; Santa Cruz Biotechnology) and Alex Fluo 594 conjugated goat anti-rat IgG secondary antibody (1:2000; Molecular Probes).

To assess the fusion with lysosomes, Texas-red conjugated ovalbumin (TroV) was used to label the lysosomes as described previously [51, 52]. Before the infection, 500 μl of pre-warmed TroV (100 μg/ml; Molecular Probes) was added to each well and incubated with cells for 0.5 h at 37 °C. Then the cells were washed 3 times for later use. The following procedures including infection, fixation, blocking, permeabilization and bacteria labeling were performed as in the Lamp-1 co-localization assay.

### Construction of *icm:gfp* fusion plasmids and fluorescence analysis

To analyze the activities of *icm* promoters, a GFP coding gene *gfpmut3*, the product of which emits intense fluorescence [56], was employed. The coding sequence of *gfpmut3* was cut out from pTLpflaG [57] with XbaI and SphI, and ligated with pJB908, yielding pGB908. Then the DNA sequences containing *icm* promoters of the 9 operon areas were amplified respectively and cloned into the multiple cloning site (MCS) upstream of *gfpmut3*, yielding pGB908X (from pGB9081 to pGB9089). The resulting plasmids were transformed into *L. pneumophila* strains by electroporation and the positive transformants were screened on thymidine-free BCYE plates.

For fluorescence analysis, *L. pneumophila* strains harboring pGB908X were inoculated into 5 ml AYE broth, and grown at 37 °C, 220 rpm for 20 h. Then the cultures were expanded into 25 ml AYE in flasks with initial optical densities (OD_600_) at approximate 0.2–0.3, and incubated to a stationary phase (OD_600_ 3.7–4.5). Subsequently 100 μl of bacteria culture was transferred into 96-well fluorometer plates for fluorescence quantification on a microplate reader (TECAN INFINITE M200) with excitation at 488 nm and emission at 540 nm. Bacteria with pGB908X were also streak inoculated on thymidine-free BCYE plates and the GFP fluorescence intensity was estimated by direct observation.

### Statistical analysis

Basic statistical analyses were performed using Excel, and one-way ANOVA was performed using SPSS followed by a post hoc Student-Newman-Keul’ s test.

## Abbreviations

CFU, Colony-forming units; *dot*, defect in organelle trafficking; GFP, green fluorescent protein; *icm*, intracellular multiplication; Lamp-1, lysosome associated membrane protein 1; LCV, *Legionella* containing vacuole; MOI, multiplicity of infection; T4BSS, type IVB secretion system; TroV, texas-red conjugated ovalbumin.

## Additional file

Additional file 1: Figure S1. Immunoblots of whole-cell bacterial extracts expressing indicated Cya hybrid proteins from the wild type (Lp02) and the *clpP* mutant (Xp02) probed with monoclonal antibody specific to the CyaA epitope (Santa Cruz Biotechnology sc-13582) and ICDH with anti-ICDH antibody (a kind gift with Dr. Vogel JP). (TIF 3783 kb)
