# Supplementary figures and images for: ClpP-deletion impairs the virulence of Legionella pneumophila and the optimal translocation of effector proteins
Source: BMC Microbiol. 2016 Aug 2;16:174. doi: 10.1186/s12866-016-0790-8 (PMC4969725; doi:10.1186/s12866-016-0790-8)

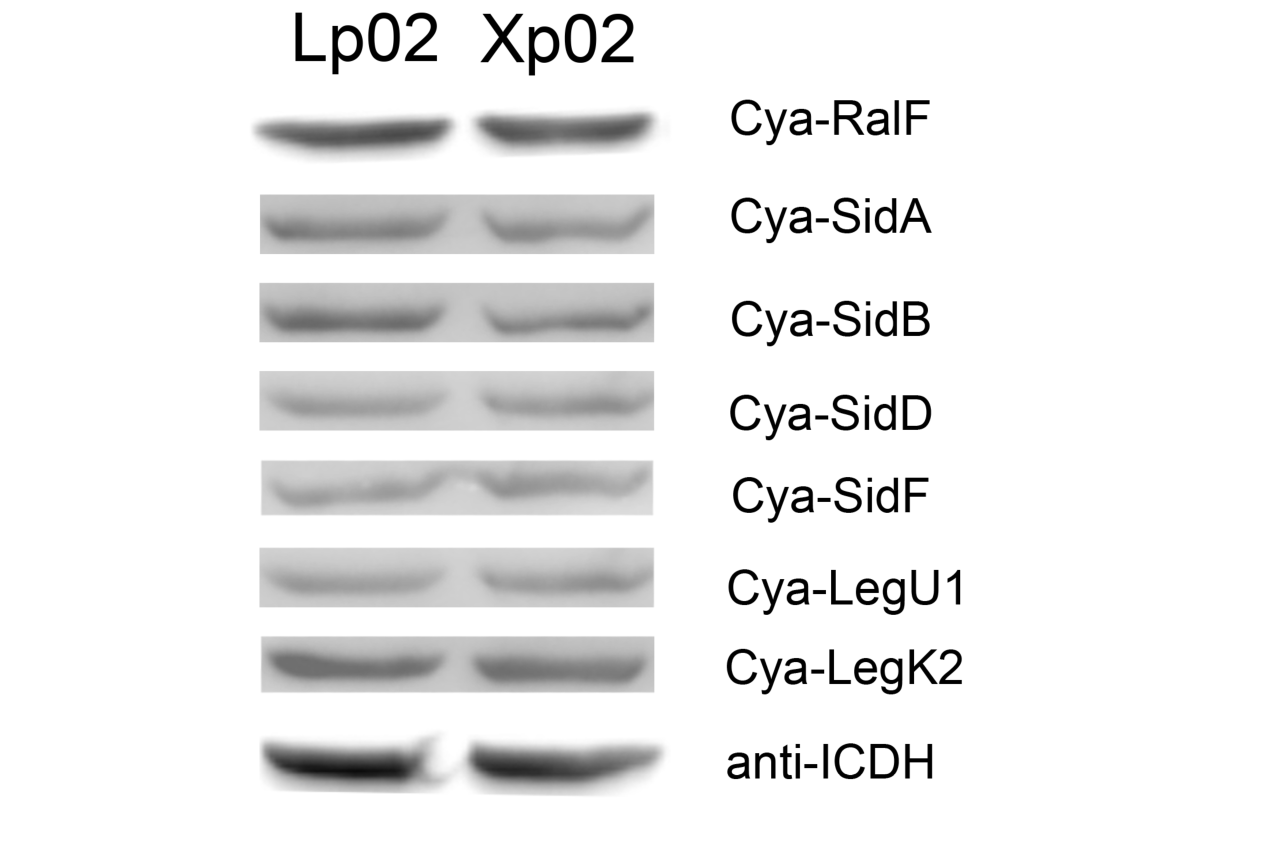

Supplement: Additional file 1: Figure S1. — Immunoblots of whole-cell bacterial extracts expressing indicated Cya hybrid proteins from the wild type (Lp02) and the clpP mutant (Xp02) probed with monoclonal antibody specific to the CyaA epitope (Santa Cruz Biotechnology sc-13582) and ICDH with anti-ICDH antibody (a kind gift with Dr. Vogel JP). (TIF 3783 kb) [file 12866_2016_790_MOESM1_ESM.tif]
